# Supplementary material for: Current State of Liver-Directed Therapies and Combinatory Approaches with Systemic Therapy in Hepatocellular Carcinoma (HCC)
Source: Cancers (Basel). 2019 Jul 31;11(8):1085. doi: 10.3390/cancers11081085 (PMC6721343; doi:10.3390/cancers11081085)
Supplement: Supplementary file 1 [file cancers-11-01085-s001.pdf]

Table S1: Selected randomized trials comparing ablation techniques. Adapted from Luo et al.*MWA, microwave ablation; RFA, radiofrequency ablation; PEI, ethanol injection; CRA, cryoablation; LSA, laser ablation.*

First author	Arms	Mean size and range (cm)	Liver recurrence rate	Survival rates
Abdelaziz et al. [93] 2014 n = 111	MWA	2.95 ± 1.03	3.9%	1 year:96% 2years:62%
	RFA	2.9 ± 0.97	13.5%	1 year:68% 2 years:47%
Shibata et al. [94] 2002 n = 72	MWA	2.3 ± 0.78[d]	1 year:10% 2 years:24%	NR
	RFA	2.2 ± 0.32	1 year:4% 2 years:12%	NR

Giorgio et al. [95] 2011 (n = 271)	PEI	2.27 ± 0.48	12.6% (18/143)	1 year: 95% 2 years:83% 3 years:78% 5 years:68%
	RFA	2.34 ± 0.45	11.7% (15/128)	1 year:95% 2 years:90% 3 years:83% 5 years:70%
Brunello et al. [7] 2008 (n = 139)	PEI	2.25 ± 0.54	63.8% (44/69)	1 year:86% 2 years:58% 3 years:25% 4 years: 7%
	RFA	2.42 ± 0.49	34.3% (34/70)	1 year:94% 2 years:59% 3 years:26% 4 years:10%
Lin et al. [96] 2004 (n = 104)	PEI	2.90 ± 0.80	1 year:23% 2 years:45% 3 years:45%	1 year:85% 2 years:61% 3 years:50%
	RFA	2.8 ± 0.8	1 year:12% 2 years:18% 3 years:18%	1 year:90% 2 years:82%

				3 years:74%
Shiina et al. [97] 2005 (n = 232)	PEI	NR	11.4% (13/114)	1 year:95%<br>2 years:82%<br>3 years:65%<br>4 years:57%
	RFA	NR	1.7% (2/118)	1 year:97%<br>2 years:92%<br>3 years:82%<br>4 years:74%
Lencioni et al. [98] 2003 (n = 102)	PEI	2.8 ± 0.8	26% (13/50)	1 year:96%<br>2 years:88%
	RFA	2.8 ± 0.6	5.8% (3/52)	1 year:100%<br>2 years:98%
Wang et al. [99] 2015 (n = 360)	CRA	NR	1 year:3%; 2 years:7%<br>3 years:7% (10/180)	1 year:97%<br>3 years:67%<br>5 years:40 %
	RFA	NR	1 year:9% 2 years:11%<br>3 years:11% (18/180)	1 year:97%<br>3 years:66%<br>5 years:38%
Di Constanzo et al. [100] 2013 (n = 140)	LSA	2.62 ± 1.04	22.9% (16/70)	1 year:94%<br>3 years:80%
	RFA	2.55 ± 0.66	25.7% (18/70)	1 year:94%<br>3 years:89%
Ferrari et al. [101] 2007 (n = 81)	LSA	2.89 ± 0.73	19.5% (8/41)	1 year:88.6%<br>2 years:70.4%<br>3 years:56.6%<br>4 years:40.2%
	RFA	2.67 ± 0.81	17.5% (7/40)	1 year:92.2%<br>2 years:75.0%<br>3 years:61.3%<br>4 years:54.6%

Table S2: Selected randomized trials comparing different embolization techniques (Adapted from Katsanos *et al.*).

cTACE, trans-arterial chemoembolization; BST, best supportive therapy; TAE, trans-arterial embolization; Y-90, trans-arterial radioembolization with yttrium-90; DEB, drug-eluting bead; OS, overall survival; PFS, progression-free survival; RR, response rate.

First author	Arms	Multinodular	Primary Endpoint	
Lo et al.[21] 2002 n=79	cTACE (cisplatin in lipiodol) vs BST	60%	3-year OS 26% 3% P<0.01	
Llovet et al.[20] 2002 n=75	cTACE (doxorubicin) vs BST	72%	2-year OS 63% 27% P<0.01	
Mabed et al.[102] 2009 n=100	cTACE (Cisplatin, doxorubicin in lipiodol) vs Doxorubicin (IV)	58%	ORR 32% 10% P<0.01	
Llovet et al. [20] (3 arm) 2002 n=72	TAE vs BST	76%	2-year OS 50% 27% P<0.01	
Raoul et al.[103] 1994 n= 27	Y-90 vs BST	70%	6-months OS 48% 0 (Zero)	
Raoul et al.[104] 1997 n= 129	Y-90 vs cTACE (cisplatin)	50%	3-year OS 22% 22% p>0.05	

Kolligs et al. [35] 2015 n= 28	Y-90 vs cTACE	68%	Quality of Life: No difference in 12 weeks	*PFS 3.6 months 3.7 months p>0.05
Salem et al. [36] 2016 n= 45	Y-90 vs TACE	47%	TTP 26 months 6.8 months P<0.01	
Lammer et al. [105] 2009 n= 201	DEB-TACE vs cTACE	42%	RR (EASL) at 6 months 52% 44% p>0.05	
Sacco et al. [106] 2011n= 67	DEB-TACE vs cTACE	43%	Complete Response at 1 month 51% 71% p NR	
Golfieri et al. [32] 2014n=177	DEB-TACE vs cTACE	54%	2-year OS 57% 55% p>0.05	
